# Human Adult Microbiota in a Static Colon Model: AhR Transcriptional Activity at the Crossroads of Host–Microbe Interaction

**DOI:** 10.3390/foods11131946

**Published:** 2022-06-30

**Authors:** Elizabeth Goya-Jorge, Irma Gonza, Pauline Bondue, Caroline Douny, Bernard Taminiau, Georges Daube, Marie-Louise Scippo, Véronique Delcenserie

**Affiliations:** 1 Laboratory of Food Quality Management, Department of Food Sciences, Faculty of Veterinary Medicine, University of Liege, Av. de Cureghem 10 (B43b), 4000 Liege, Belgium; egoya@uliege.be (E.G.-J.); iegonza@uliege.be (I.G.); 2 Research & Development, ORTIS S.A., Hinter der Heck 46, 4750 Elsenborn, Belgium; pauline.bondue@ortis.com; 3 Laboratory of Food Analysis, Department of Food Sciences, Faculty of Veterinary Medicine, University of Liege, Av. de Cureghem 10 (B43b), 4000 Liege, Belgium; cdouny@uliege.be (C.D.); mlscippo@uliege.be (M.-L.S.); 4 Laboratory of Microbiology, Department of Food Sciences, Faculty of Veterinary Medicine, University of Liege, Av. de Cureghem 180 (B42), 4000 Liege, Belgium; bernard.taminiau@uliege.be (B.T.); georges.daube@uliege.be (G.D.)

**Keywords:** AhR agonism, aryl hydrocarbon receptor, biogenic amines, gastrointestinal simulation, human gut microbiota, intestinal metabolites, intestinal microbial community, in vitro fermentation, host–microbe interaction, reporter gene assays

## Abstract

Functional symbiotic intestinal microbiota regulates immune defense and the metabolic processing of xenobiotics in the host. The aryl hydrocarbon receptor (AhR) is one of the transcription factors mediating host–microbe interaction. An in vitro static simulation of the human colon was used in this work to analyze the evolution of bacterial populations, the microbial metabolic output, and the potential induction of AhR transcriptional activity in healthy gut ecosystems. Fifteen target taxa were explored by qPCR, and the metabolic content was chromatographically profiled using SPME-GC-MS and UPLC-FLD to quantify short-chain fatty acids (SCFA) and biogenic amines, respectively. Over 72 h of fermentation, the microbiota and most produced metabolites remained stable. Fermentation supernatant induced AhR transcription in two of the three reporter gene cell lines (T47D, HepG2, HT29) evaluated. Mammary and intestinal cells were more sensitive to microbiota metabolic production, which showed greater AhR agonism than the 2,3,7,8-tetrachlorodibenzo-p-dioxin (TCDD) used as a positive control. Some of the SCFA and biogenic amines identified could crucially contribute to the potent AhR induction of the fermentation products. As a fundamental pathway mediating human intestinal homeostasis and as a sensor for several microbial metabolites, AhR activation might be a useful endpoint to include in studies of the gut microbiota.

## 1. Introduction

The human gastrointestinal tract hosts a plethora of microorganisms engaged as a functional entity. In fact, the total number of gut bacteria is estimated to be close to the total count of cells in our bodies [1]. The three domains of life, Eukarya, Bacteria, and Archaea [2], as well as their viruses, are included in the intestinal microbiome as a complex ecological community [3]. The gut microbiome has its own physiology and pathology, and increasing evidence supports its multiple functions (beneficial or detrimental) [4]. However, whereas some suggest considering the gut microbiota a “virtual organ” or “emergent system” [5,6], many others remain cautious to describe it as such [7]. In any case, a constantly growing body of evidence is bringing us closer to understanding host–microbe species associations and the consequences of being interdependent meta-organisms [8].

The host response to xenobiotics is an important function long linked to the gut microbiome [9]. With the acknowledgment of xenobiotics sensors, such as the aryl hydrocarbon receptor (AhR), as direct mediators of the host–microbiota interplay, a greater understanding of this process has arisen [10,11]. From its first discovery, AhR (for a long time called the “dioxin receptor”) was related to the complex machinery involved in detoxification responses and understood as a sensor of industrial byproducts such as dioxin-like compounds (i.e., 2,3,7,8-tetrachlorodibenzo-*p*-dioxin (TCDD), dibenzofurans, biphenyls) [12], as well as mixtures of persistent organic pollutants [13]. Indeed, the intestinal mucosa is the portal to external stimuli (e.g., toxicants, nutrients), and AhR transcriptional activity is one of the host strategies for responding to such exposures [10].

The intestinal microbiota also provides a wide range of metabolically active molecules that in many cases cannot be produced by the host. The ubiquitous AhR is capable of processing several of these chemical signals despite their structural diversity [14,15]. However, the mechanisms underpinning such AhR–microbiota communication are complex and liable to be refined. What is clear is that some gut fermentation products such as short-chain fatty acids (SCFA) and L-tryptophan catabolites are able to activate AhR and, through this pathway, they have an important impact on human physiology and health [14,16]. 

A bidirectional AhR–microbiome axis allows for the control of host intestinal homeostasis [17], and AhR activation has a fundamental role in host defense against diverse pathogenic threats [18,19,20]. Further, the receptor contributes to the maintenance of gut epithelial barrier integrity through the control of key pathways such as the formation of intestinal lymphoid follicles [21], which act in the regular regeneration of the colon mucosa [22]. 

By up-regulating the production of IL22, AhR protects intestinal stem cells from genotoxicants and contributes to gut epithelial regeneration [23,24,25]. Thus, an intestinal deficiency of AhR is associated with an increase in gut epithelial immunopathology. Mice models have shown severe symptoms (i.e., extreme shortening of the colon, accelerated weight loss, and hemorrhages) in the absence of AhR transcriptional activity [26]. 

Moreover, several naturally occurring and natural-mimic ligands of AhR are chemotherapeutic suggestions for human colorectal cancer [27,28,29]. 

As a crucial gut immunomodulator, AhR is highly expressed in macrophages, dendritic cells, and T cells (e.g., FoxP3^+^ Treg, Tr1, Th17, Th22) [16,17,30]. In dendritic cells, AhR participates in the expression of the enzyme indoleamine 2,3-dioxygenase (IDO), which is involved in tryptophan metabolism [31]. Furthermore, AhR activation promotes the transdifferentiation of Th17 into Tr1 [32], particularly important for regulating intestinal inflammation [33]. In systemic autoimmune disorders such as rheumatoid arthritis, the immunosuppressive function of regulatory B cells has been linked to the AhR transcriptional program [34].

Collectively, these findings identify AhR as a model signaling pathway to investigate the molecular mechanisms through which the microbiota-derived metabolites might control host immune and inflammatory responses in both health and disease. 

In this work, the AhR activation caused by the metabolic output of the gut fermentation process in a short-term (72 h) and static in vitro simulation of the human colon was studied. Ultimately, we hope this analysis will provide valuable insights to motivate the study of complex mixtures of microbiota-derived metabolites on AhR transcriptional pathways as a way to better assess host–microbe interactions.

## 2. Materials and Methods

Chemical reagents were obtained from Sigma Aldrich (Merck KGaA, Darmstadt, Germany) and consumable materials from Greiner Bio-One BioScience (Vilvoorde, Belgium) unless otherwise specified. The full names of suppliers are declared on the first mention only. 

### 2.1. Reagents and Biological Material

#### 2.1.1. Human Fecal Material 

Research with human fecal material was approved by the Ethical Committee of the Liège University-Hospital (file number 2020/293). The three healthy adult donors were all female, ranging in age from 33 to 44 years old. They all met the following criteria: had a normal body mass index (BMI < 30), consumed a typical western diet (not vegan or vegetarian), and were antibiotic-free for at least 6 months before feces recollection. 

Collected fecal samples were immediately stored at 4 °C inside anaerobic jars using Anaerogen™ 3.5 L bags (Oxoid, Basingstoke, UK). Once in the lab (maximum delay of 3 h), feces were mixed with a phosphate buffer solution containing 8.8 g of K_2_HPO_4_, 6.8 g of KH_2_PO_4_, and 0.1 g of sodium thioglycolate in 1 L of demineralized water. Approximately 20 g of this suspension was placed in a double-coated sterile stomacher bag (300 × 190 mm) with a lateral filter, and enough buffer was then added to reach a content of 20% (*w*/*v*) of feces in the suspension. The mixture was homogenized using a Stomacher VWR^®^ Star-Blender LB400 for 10 min at 2 min intervals. Finally, filtered homogenates were mixed with 20% glycerol as a cryoprotectant (*v*/*v*) [35] and stored at −80 °C until inoculation. 

#### 2.1.2. Human Cell Lines in Culture

The AhR activation was evaluated using three human AhR-reporter gene cell lines (AhR_T47D, AhR_HepG2, AhR_HT29-Lucia) derived from mammary, hepatic, and intestinal tissues, respectively. The AhR_T47D and AhR_HepG2 cell lines used are two home-made stably transfected cells [36]. AhR_T47D cells were grown in DMEM (Dulbecco’s Modified Essential Medium), and AhR_HepG2 cells were grown in MEM (Minimum Essential Medium). Both culture media were supplemented with 10% heat-inactivated fetal bovine serum (FBS) and 100 μg/mL of an antibiotic mixture of Penicillin-Streptomycin (Pen/Strep). 

Colon adenocarcinoma AhR_HT29-Lucia cells were obtained from Invivogen (Toulouse, France) and cultured according to the provider’s instructions in DMEM supplemented with 4.5 g/L glucose, 2 mM L-glutamine, 10% of FBS, and antibiotics including: 100 μg/mL Pen/Strep, 100 μg/mL of Normocin, and 100 μg/mL of Zeocin. 

All cell lines were incubated in 75 cm^2^ culture flasks at 37 °C in 5% CO_2_. The growth medium was regularly renewed, and weekly passages upon 80–90% confluency were conducted by rinsing cell layers with phosphate-buffered saline solution (PBS, 1X, pH 7.4) and gently detaching the cells using a 0.25% solution of trypsin-EDTA. 

### 2.2. Human Colon Simulation Set-Up

A simplified screening alternative of the pH-controlled, automated SHIME^®^ technology (ProDigest^®^, Ghent, Belgium) was used to simulate the human colonic ecosystem (referred here as static or “batch” culture experiment) over 72 h. In this work, the short-term colonic simulation was conducted in triplicate, allowing for parallel comparison of the fermentation process. Stool samples from three donors (1, 2, and 3) were pooled in equal proportions (*v/v/v*) to prepare the fecal inoculum [37]. 

Double-jacketed vessels containing 600 mL of nutritional media (Prodigest, Ghent, Belgium) were inoculated with 30 mL of fecal material. This growth media is specially formulated with ingredients normally available for fermentation in the human adult colon, including arabinogalactan (1.2 g/L), pectin (2.0 g/L), xylan (0.5 g/L), glucose (0.4 g/L), yeast extract (3.0 g/L), special pepton (1.0 g/L), mucin (3.0 g/L), L-cystein-HCl (0.5 g/L), and starch (4.0 g/L). The mixture was diluted in distilled water (15.6 g/L), vigorously shaken, and autoclaved (121 °C) 24 h before use.

The temperature in the vessels was maintained at 37 °C using a warm water circulation, and all reactors were in continuous agitation (300 rpm). The vessels were closed airtight to maintain anaerobiotic conditions, and the headspace was flushed once a day with nitrogen gas (N_2_ flow threshold = 2.0 L/min).

The pH during the three days of fermentation was automatically maintained between 6.6–6.9 via the pump-regulated addition of NaOH (0.5 M) and HCl (0.5 M) (ChemLab, Zedelgem, Belgium). Moreover, daily checks of the pH values inside the reactors were conducted via an external pH meter (Mettler-Toledo, Zaventem, Belgium). The online monitoring of the acid and base consumption (mL/day) was recorded as an indicator of microbial activity. 

### 2.3. Analysis of Gut Microbial Composition 

#### 2.3.1. DNA Extraction from Fermentation Samples

A volume of 2 mL of each freshly harvested fermentation sample (from day 0 to day 3) was used to obtain ~200 mg of bacterial cells in a pellet by centrifugation (17,000× *g*, 5 min, at room temperature) using a Microcentrifuge MicroStart17 from VWR^®^. These pellets were stored at −20 °C until DNA extraction.

Bacterial DNA was extracted using the PSP^®^ Spin Stool DNA Kit (Invitek Molecular GmbH, Berlin, Germany) following the manufacturer’s instructions. Briefly, bacteria were lysed by a 10-min incubation at 95 °C under shaking (900 rpm) on a thermomixer (Eppendorf, Aarschot, Belgium). After several purification steps, samples were incubated with Proteinase K for 10 min at 70 °C under shaking (900 rpm) and 200 µL of binding buffer was added to the lysate. The mixture was transferred to the spin column that bound the nucleic acids, and residual contaminants were removed by several washing steps. The elution of 200 µL of DNA from the membrane was made possible by adding a preheated (70 °C) low salt buffer. The DNA concentrations yielded were measured using a Nanodrop 2000 spectrophotometer (Thermo Fisher Scientific, Waltham, MA, USA). The quality and purity of the extracted DNA were estimated using the 260/280 nm and 230/260 nm ratios.

The extracted DNA was diluted to reach proper concentrations for gut microbial composition analyses through 16S rRNA sequencing and quantitative polymerase chain reaction (qPCR) experiments, indistinctly.

#### 2.3.2. 16S Amplicon Sequencing and Microbiota Profiling

PCR amplification of the microbial V1-V3 regions of 16S rRNA gene and sequencing library preparation were performed with the forward 5′-GAGAGTTTGATYMTGGCTCAG-3′ and reverse 5′-ACCGCGGCTGCTGGCAC-3′ primers using Illumina^®^ technology overhand adapters. An Agencourt AMPure XP beads kit (Beckman Coulter, Brea, CA, USA) was used for the purification of PCR products. Then, the second round of PCR for indexing was developed with Nextera XT Index Kit followed by purification. A fluorescence assay with Quant-iT™ PicoGreen dsDNA Quantitation Reagent (Invitrogen, Waltham, MA, USA) was developed to prepare dilutions of 10 ng/μL of DNA. Finally, qPCRs were conducted using KAPA SYBR^®^ FAST Kit (KapaBio, Wilmington, MA, USA), followed by normalization, pooling, and Illumina^®^ MiSeq-based high throughput amplicon sequencing of the 16S rRNA microbial gene to identify microbial communities in fecal contents (Illumina, San Diego, CA, USA).

The processing of Next Generation Sequencing (NGS) data included the alignment, clustering (distance = 0.03) in operational taxonomic units (OTU), calculation of the sequencing coverage, as well as the assessment of biodiversity through four estimators (i.e., Shannon index, Chao richness, reciprocal Simpson index, and evenness), all carried out on a rarefied table (10,000 read per sample) using MOTHUR software package v1.39 (https://www.mothur.org) (accessed on 22 February 2022). For the removal of chimeric sequences, the VSEARCH algorithm (https://github.com/torognes/vsearch) (accessed on 8 March 2022) was used [38]. The taxonomical assignment was conducted using the SILVA release 138 reference database (https://www.arb-silva.de) (accessed on 8 March 2022) [39]. Within each sample, OTU counts were divided by the total OTU count to obtain the relative abundances as a percentage.

#### 2.3.3. qPCR Analysis of Selected Targets

qPCR was performed using Takyon™ No ROX SYBR MasterMix and the forward and reverse primers of each taxa-specific population (Eurogentec S.A., Seraing, Belgium). Reactions were performed in hard-shell 96 well plates (Bio-Rad Laboratories Inc., Tense, Belgium) covered with AMPLIseal™ plate sealers. The C1000™ Touch Thermal Cycler and CFX Maestro™ software v.4.1 from Bio-Rad were utilized as detection systems. 

All qPCR reactions contained 10 μL of the MasterMix and the pair of primers (from 0.3–0.5 µM) diluted in molecular biology grade water to reach 17.5 μL. A volume of 2.5 µL of each DNA template (~4 ng/μL) was added to reach a final volume of 20 μL per well. A Non-Template Control (NTC) was included in all assays. Sealed plates were briefly centrifuged (3500 g, 1 min, at room temperature) before being loaded into the thermocycler. 

A single qPCR protocol was validated according to standard guidelines for fluorescence-based qPCR experiments for all taxon targets analyzed, variating only the optimal annealing temperature (Tann) (Supplementary File S1, Table S1). The cycling protocol included an initial denaturation step at 95 °C for 5 min, 35 cycles at 95 °C for 15 s, the corresponding Tann for 15 s, and 72 °C for 30 s, followed by a final elongation step at 72 °C for 5 min [40]. Fluorescent data were obtained during the extension phase.

The analysis of the melt curves (65 °C–95 °C) allowed us to estimate the specificity of the amplified products. Primer’s efficiency was inferred from the shift of the quantification cycle (Cq) of an eight-point calibration curve designed with mixed DNA (from 0.025 ng–40 ng) from all the fermentation experiments. Efficiency percentages were validated to be between 90–105%, and regression coefficients above 0.98, following the recommendations of MIQE guidelines [41].

A comparative quantification via the 2^−ΔΔCq^ method was used to report the expression levels of the target populations [42]. Thus, results are stated as a fold increase (or decrease) of the target in the samples relative to the calibration sample (Day 0—‘inoculation’), and they were normalized to the expression of the reference sequence used as ‘universal’ (total) bacteria.

### 2.4. Chromatographic Analysis of Gut Microbiota-Derived Metabolites 

#### 2.4.1. Short-Chain Fatty Acids by SPME-GC-MS

An analytical method using solid-phase microextraction (SPME) followed by gas chromatography coupled to mass spectrometry (GC–MS) was utilized for the SCFA quantification, as previously validated [43]. A Triplus RSH Auto-sampler (Thermo Fisher Scientific, Waltham, MA, USA) with a SPME fiber DVB/CAR/PDMS (Supelco, Bellefonte, PA, USA) was set up for SCFA extraction (20 min) with an agitation temperature of 60 °C and desorption (5 min) at 250 °C. Fiber conditioning post-injection was performed for 10 min at 270 °C, and the separation was completed in a Supelcowax-10 (30 m × 0.25 mm, 0.2 μm) column (Supelco) and analyzed with an ion trap PolarisQ mass spectrometer (Thermo Fisher Scientific). Peaks were identified by comparing mass spectra and retention times with those of the corresponding standards. The concentrations of acetic (C2), propionic (C3), isobutyric (iC4), butyric (C4), isovaleric (iC5), valeric (C5), and caproic (C6) acids in the batch experiments were thereby determined in a single run.

Analyzed samples contained 40 μL of 2-methylvaleric acid (0.2 mg/mL) in water as an internal standard, 15 μL of sulfuric acid (0.9 M), 920 μL of water, and 25 μL of the harvest fermentation samples (without any previous centrifugation or filtration). The final volume of 1 mL (pipetted into a 20 mL glass vial) was vigorously vortexed before analysis in the SPME-GC-MS system. 

The SCFA limits of quantification (LOQ) were the following: 2.40–119.90 mM (C2), 1.09–54.67 mM (C3), 0.79–39.72 mM (C4), 0.47–23.50 mM (C5), and 0.02–0.86 (C6). For BCFA, the LOQs were between 0.18–9.08 mM and 0.14–6.85 mM for iC4 and iC5, respectively. 

#### 2.4.2. Biogenic Amines by UPLC-FLD

The preparation of fermentation samples and derivatization were adapted from a previous work [44]. To 500 μL of the samples were added 25 μL of the internal standard solution (1,7-diaminoheptane (100 ng/μL) prepared in trichloroacetic acid 5%). Then, 475 μL of 0.4 M of perchloric acid were added, vortexed for 20 s, and centrifuged (17,746 g, 5 min, at room temperature). This extraction was performed twice, and both supernatants were combined. One milliliter was then transferred into a 15-mL Falcon tube, and 200 μL NaOH (2 N) and 300 μL of saturated NaHCO_3_ were added, vortexing the tube after each addition. The dansylation was realized by adding 2 mL dansyl chloride (10 mg/mL in acetone), followed by incubation for 15 min at 70 °C. A volume of 100 μL of glycine (150 mg/mL in water) was then added to bind to the dansyl chloride in excess, followed by vortex and a second incubation of 15 min at 70 °C. Finally, samples were centrifuged (5 min, 3700 g at room temperature) and maintained at 5 °C until 5 μL of the final extract were injected into the system for analysis.

UPLC-Fluorescence Analysis. Biogenic amines were analyzed in an Acquity system ultra-performance liquid chromatography with fluorescence detection (UPLC-FLD), using the Acquity UPLC BEH C18 (2.1 × 100 mm, 1.7 μm) column, with a UPLC BEH C18 VanGuard pre-column (2.1 × 5 mm, 1.7 μm), all from Waters Corporation (Milford, MA, USA), using previously validated conditions [44]. Thus, the concentrations of nine amines (i.e., tryptamine, tyramine, cadaverine, spermine, spermidine, 2-phenylethylamine, putrescine, histamine, methylamine) in the fermentation samples were determined in a single run. The LOQ ranged between 8.4 mM and 314.5 mM for histamine and between 0.002 and 1.986 mM for the rest of the biogenic amines.

### 2.5. Cell Culture Experiments

#### 2.5.1. Treatments and Controls

Fermentation samples (stored at −20 °C) were defrosted for ~1 h and centrifuged at 9500 rpm. The supernatants were collected and sterilized using Whatman^®^ syringe filters (0.2 μm). Two different doses of filtered, sterilized supernatant from the fermentation samples were used to evaluate AhR activation as follows:

- Dose 1: 20 µL of fermentation sample + 30 µL of the fermentation medium.
- Dose 2: 50 µL of fermentation sample.

Both treatments were mixed with antibiotic free-cell culture medium before adding them to overnight-seeded cells (~2.0 × 10^5^ cells/mL). Validated calibration curves (from 0.025–10 nM) of the standard agonist control TCDD with a final DMSO concentration of 0.4% (*v/v*) were incorporated into all experiments for comparison and as quality control of the assays. Cells exposed to the fermentation medium and to the TCDD solvent dimethyl sulfoxide (DMSO), as well as untreated cells, were included in all plates.

#### 2.5.2. AhR Activity Assay 

Two protocols of AhR reporter gene assays (RGA) were developed depending on the studied cell line.

(a) AhR_T47D and AhR_HepG2 cells were seeded (3.0 × 10^5^ cells/mL) and incubated overnight in white clear-bottomed 96-well plates, and later exposed to the fermentation samples for 24 h. For the luminescence reading, cells were rinsed with PBS, and 50 µL/well of lysis solution containing Triton X100 were added. The plates were frozen at −20 °C for two hours to boost the lysis, and finally, 50 µL/well of glow-mix containing luciferin (Promega, Madison, WI, USA) and ATP (Roche Diagnostics, Rotkreuz, Switzerland) were added.
(b) AhR_HT29 Lucia cells were seeded (3.0 × 10^5^ cells/mL) in CellStart^®^ 96-well microplates and incubated overnight before treatment. After 24 h of exposure to the fermentation samples, 20 µL of the cell supernatant were transferred to Nunc™ white 96-well plates. Then, 50 µL/well of Quanti-Luc™ assay reagent were added.

Bioluminescence for both assay protocols was determined using a luminometer (ORION II, Berthold Detection System, Pforzheim, Germany). AhR-RGA experiments were conducted in triplicate for all samples from the three independent replicates of the colon simulation (n = 9). The signals quantified in relative light units (RLU) were expressed as a fold induction of the AhR transcription of treated cells relative to nontreated cells.

#### 2.5.3. Cytotoxicity Analysis

Cell viability was studied upon treatment in the three cell lines following the 3-(4,5-dimethylthiazol-2-yl)-2,5-diphenyltetrazolium bromide (MTT) bioassay, which evaluates the reduction capacity of metabolically active cells [45]. For AhR-HT29 Lucia, the same protocol used for a different Lucia luciferase cell line was followed [46]. This allowed for the simultaneous assessment of the AhR activity, and the potential cytotoxicity caused by the treatments. For AhR-T47D and AhR-HepG2 cell lines, the cytotoxicity was studied as detailed previously [47]. The MTT formazan absorbance was read in all cases at 550/630 nm using an ELX800TM microplate reader spectrophotometer (Agilent BioTek Inc., Winooski, VT, USA). In parallel, routine inspections of cell morphology and attachment were conducted to detect any sign of contamination by visual inspection under the inverted microscope and through a cell counting on passages using a Countess™ 3 Automated Cell Counter (Invitrogen). 

### 2.6. Statistical Analysis 

Data are expressed as means ± SD of at least three independent experiments, and comparisons were performed with a Student’s *t*-test or using one-way analysis of variance (ANOVA) followed by Bonferroni’s post-hoc test. Differences were considered statistically significant if *p* values were <0.05. All statistical comparisons and graphical representations were performed using GraphPad Prism version 9.0.1 for macOS, GraphPad Software (San Diego, CA, USA).

## 3. Results

### 3.1. The Microbial Composition of the Inoculum from Each Donor

**Figure 1:**
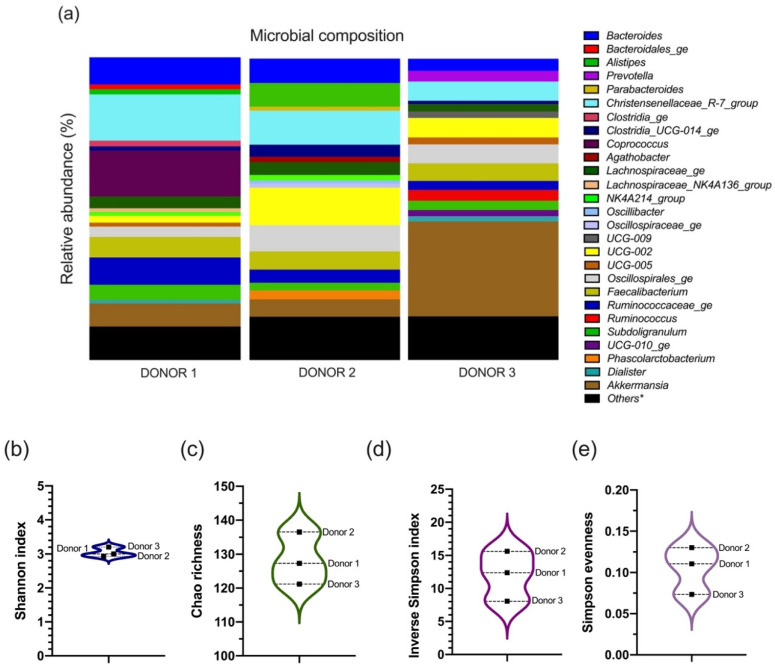
Microbial community analysis in stool samples from donors 1, 2, and 3, as assessed using 16S rRNA Illumina Sequencing. (**a**) Genera abundance histogram (%, left panel) and genus classification (right panel) based on quality-controlled OTU reads. Only bacterial genera with relative abundance >1% in at least one sample are shown, and the rest are included as others * (see details in Supplementary File S2, Table S2). Diversity analysis of the sequencing data is presented as (**b**) Shannon α-diversity index, (**c**) Chao estimator of genus richness, (**d**) Simpson reciprocal α-diversity index, and (**e**) Simpson abundance-derived evenness.

The analysis of the microbiota composition in the inoculum through 16S rRNA sequencing is shown in Figure 1.

Sequencing coverage of the stool samples at the genus level reached 0.9976, 0.9978, and 0.9980 values for donors 1, 2, and 3, respectively. The highest number of unique genera was identified in donor 2 with 120. Donors 1 and 3 had a similar count of unique genera, with 112 and 110, respectively, of which 77 genera were common to both donors. Overall, inter-individual differences in genera abundance were observed between the three donors, as displayed in Figure 1a.

Various metrics were calculated to estimate the diversity within the bacterial community of each fecal material sample at the genus level. The Shannon alpha-diversity index (Figure 1b) did not reveal any difference in the gut bacteria population’s diversity of the three donors. The inverse Simpson (Figure 1d) biodiversity index and population evenness (Figure 1e), as well as the Chao richness estimator (Figure 1c), suggested a slightly higher diversity for donor 2, followed by donor 1, and, lastly, donor 3.

### 3.2. Evolution of Target Bacteria during the 72-h Fermentation

**Figure 2:**
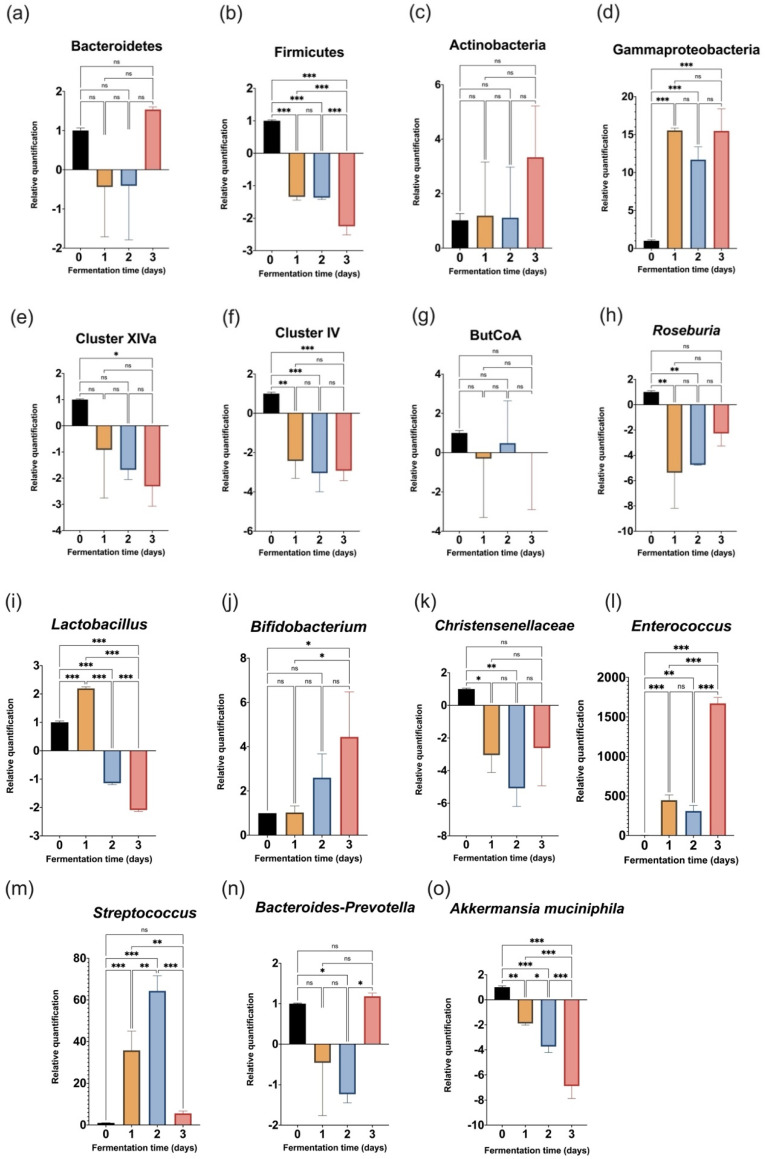
Relative quantification of fifteen target taxa assessed by qPCR analysis. Data are presented as mean ± SD fold-changes calculated using 2^−ΔΔCq^ method [42]. Comparisons were performed by means of one-way ANOVA, followed by Bonferroni post-test, and statistical significance is indicated as *p* < 0.05 (*), *p* < 0.01 (**), *p* < 0.001 (***), and *p* > 0.05 (ns).

In order to monitor the evolution of fifteen target bacterial taxa during the three days of colonic fermentation, qPCR analyses were conducted. Thus, Figure 2 presents, indistinctly, the results of the microbial community composition analysis for Bacteroidetes, Firmicutes, and Actinobacteria phylum, as well as for the *Gammaproteobacteria* class, the communities *Bacteroides-Prevotella*, *Clostridium coccoides* (Cluster IV), *Clostridium leptum* (Cluster XIVa), and butyrate-producing bacteria based on the detection of butyryl-coenzyme A transferase genes (ButCoA). The *Christensenellaceae* family, some selected genera, and the *Akkermansia muciniphila* species are also represented. Changes over time were analyzed independently using the daily harvested fermentation samples. 

The Firmicutes phylum (Figure 2b) and *Akkermansia muciniphila* species from the Verrucomicrobia phylum (Figure 2o) both decreased during the fermentation, and such diminution was significantly higher by the third day of the experiment. In parallel, levels of the Gammaproteobacteria class and the *Enterococcus* genus significantly increased during the three days of colonic fermentation, as shown in Figure 2d,l, respectively. 

The *Streptococcus* genus increased significantly during the first and second day (Figure 2m). However, by the third day, *Streptococcus* levels returned to values not significantly higher than those present initially (Day 0). The analyzed ButCoA genes did not reveal any important changes in butyrate-producing bacteria (Figure 2g).

The *Roseburia* genus (Figure 2h), as well as the Cluster IV group (Figure 2f) that includes important genera (e.g., *Ruminococcus*, *Faecalibacterium*, *Oscillibacter*, etc.), were significantly reduced during the experiment. The Cluster XIVa group (Figure 2e) decreased, but only at significant levels on the last day. A significant decrease in the *Christensenellaceae* family was identified in fermentation samples after the first and second days of the simulation (Figure 2k).

The *Lactobacillus* genus increased during the first day of incubation, but after that, it significantly decreased (Figure 2i). In contrast, *Bacteroides*-*Prevotella* (Figure 2n) and its phylum Bacteroidetes (Figure 2a) were identified only by the end of the fermentation (third day). However, these last two taxa were not quantified at statistically important levels when compared with the fecal inoculum.

Finally, the levels of the Actinobacteria phylum did not have any significant variation (Figure 2c) during the experiment. Nevertheless, the *Bifidobacterium* genus increased on the third day with respect to the day of inoculation and the first day after inoculation (Figure 2j).

### 3.3. Colon Microbiota-Derived Metabolic Production 

The bacterial fermentation in the in vitro colonic systems was evaluated through the chromatographic quantification of metabolites (i.e., SCFA, branched-chain fatty acids (BCFA) and biogenic amines) in daily collected samples.

#### 3.3.1. Volatile Fatty Acids Profile 

**Figure 3:**
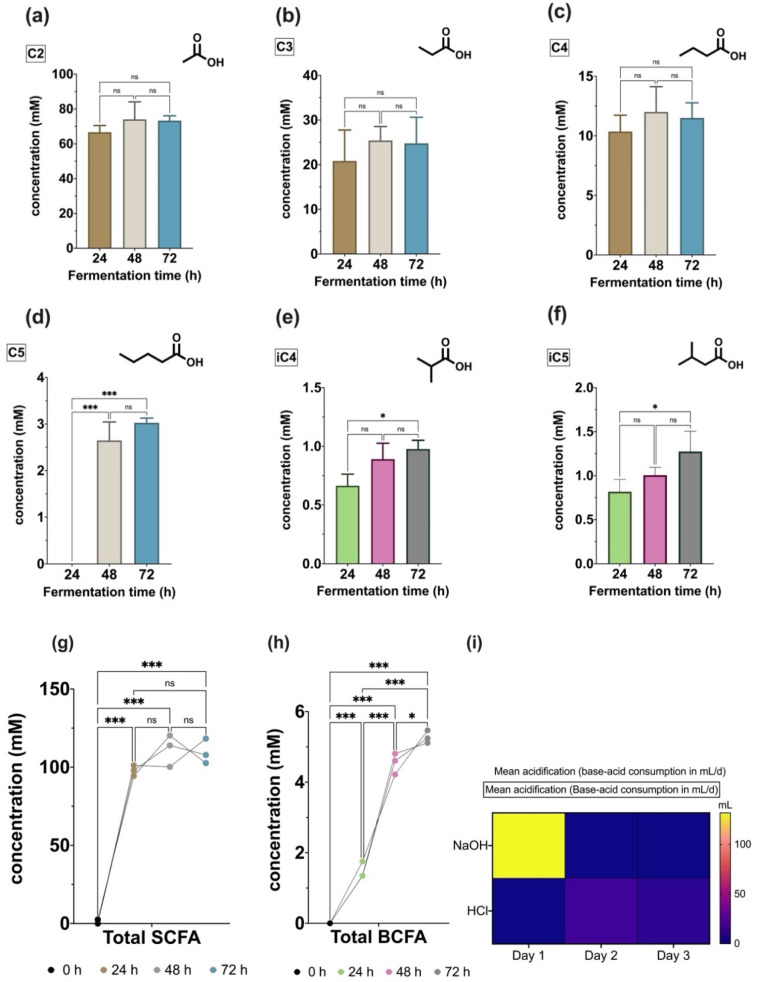
Production of short-chain fatty acids (SCFA) and branched-chain fatty acids (BCFA) and acidification during the in vitro fermentation. Concentrations are expressed in mM for: (**a**) C2: acetate, (**b**) C3: propionate, (**c**) C4: butyrate, (**d**) C5: valerate, (**e**) iC4: isobutyrate, (**f**) iC5: isovalerate, (**g**) total quantification of SCFA, and (**h**) BCFA, all quantified using SPME-GC-MS. (**i**) Recorded consumptions (mL/day) of base (NaOH) and acid (HCl). Data are presented as mean ± SD, and statistical significances (one-way ANOVA comparisons, followed by Bonferroni’s post-test) are shown as *p* < 0.05 (*), *p* < 0.001 (***), and *p* > 0.05 (ns).

Figure 3 shows the metabolic production of SCFA and BCFA obtained in the model and the acidification (mL/day of acid/base consumption) automatically registered by the system during the 3 days of colonic fermentation.

The production of C2, C3, and C4 was identified from the first 24 h of fermentation and remained at constant levels after that. The highest concentrations were obtained for acetic acid, with values between 60–80 mM (Figure 3a). Propionic acid was quantified between 20–40 mM (Figure 3b), and butyric acid was found between 10–15 mM in the fermentation samples (Figure 3c). Valeric acid (C5) was detected (~2–3 mM) only after the second day of fermentation (Figure 3d), after which it remained relatively constant. Caproic acid (C6) was not detected in the simulated colon experiment. Consistent with the production of individual SCFA, the total production of SCFA (Figure 3g) was only significantly higher when compared with the inoculation day (0 h). However, total SCFA production remained relatively invariable during the 72-h fermentation (~100 mM).

Branched-chain fatty acid (BCFA) production was time-dependent. Hence, significant increases in the total production of these metabolites were quantified every 24 h (Figure 3h). However, the individual production of isobutyrate (iC4) and isovalerate (iC5) significantly increased only on the third day of fermentation, as observed in Figure 3e,f, respectively. 

Finally, the registered consumption of the base (NaOH) and acid (HCl) during the simulation (Figure 3i) showed a high demand for the basic solution during the first 24 h. Nonetheless, by the second and third day of fermentation, no important consumptions of the base or acid were necessary to maintain the in vitro colon with a pH between 6.6–6.9.

#### 3.3.2. Biogenic Amines Profile 

**Figure 4:**
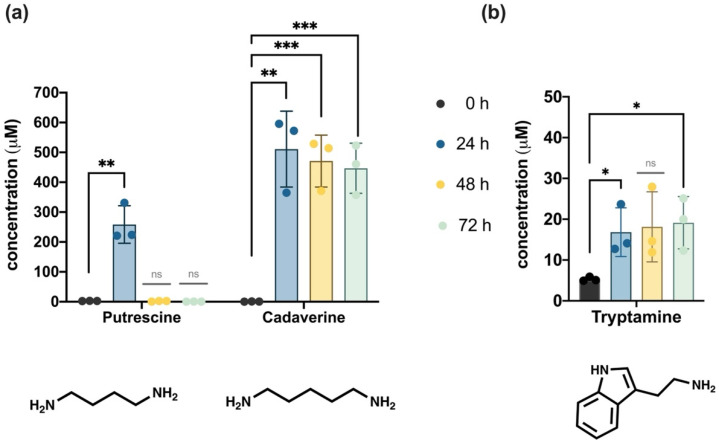
Microbiota-derived production of (**a**) putrescine and cadaverine and (**b**) tryptamine. Data are expressed as mean ± SD. Statistically significant differences evaluated using one-way ANOVA comparisons followed by Bonferroni’s post-test are indicated as *p* < 0.05 (*), *p* < 0.01 (**), *p* < 0.001 (***), and *p* > 0.05 (ns).

The colon microbiota-derived production of nine biogenic amines was analyzed. The measured concentrations of the two polyamines, putrescine and cadaverine, and the tryptophan metabolite tryptamine, are presented in Figure 4a,b, respectively.

As shown in Figure 4a, cadaverine was abundantly produced (~400–600 µM) by the intestinal microbiota. The structural polyamine analogous putrescine was detected above 200 µM, but only in samples from the first day of fermentation. In addition, between 10–30 µM of tryptamine were produced by the microbiota after 24 h and 72 h (Figure 4b), which was significantly higher than the concentration observed at the time of the inoculation.

The results of the UPLC-FLD quantification of the remaining six biogenic amines (i.e., methylamine, spermidine, spermine, 2-phenylethylamine, tyramine, histamine) are shown in Table 1. 

After 48 h of fermentation (see Table 1), a concentration of 35.75 µM of methylamine was detected in one of the replicates. By the end of the colonic simulation, greater levels of methylamine were identified in all samples, yet with a very high standard deviation between replicates. The polyamine spermidine was identified at the time of the inoculation (0 h), and after 48 h of the experiment, at concentrations lower than 1 µM, but in one of the technical replicates only. Its derivative spermine was not detected in the fermentation samples. The monoamine neurotransmitter histamine was exclusively detected in one of the replicates at 0 h. 

After 24 h, and until the end of the colonic fermentation, productions of the aromatic amines 2-phenylethylamine and tyramine were identified. Though concentrations of 2-phenylethylamine ranged between 3–5 µM, tyramine was produced in greater concentrations. However, important variations in tyramine production were identified between replicates.

### 3.4. Microbiota-Derived Metabolites Activate the AhR Transcription 

**Figure 5:**
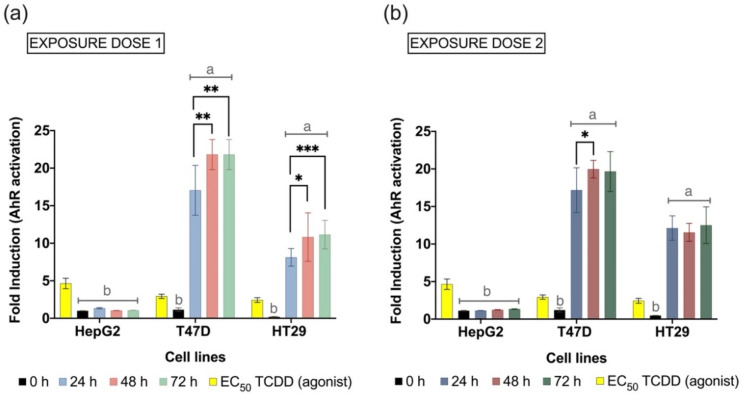
AhR transcriptional activity induced by the metabolic output samples and the agonist control TCDD. The three AhR-reporter gene cell lines (HepG2, T47D, HT29) were exposed in triplicate to the samples from the three independent repetitions of the colon simulation (n = 9). Results are expressed as mean fold induction ± SD of luminescence prompted by the exposed cells when compared with untreated cells in the same assay plate. (**a**) Exposure dose 1:20 µL of sample + 30 µL of fermentation medium, (**b**) exposure dose 2:50 µL of sample. Treatments corresponded to filtered, sterilized supernatant harvest from the colonic fermentation at the inoculation day (0 h) and after 24 h, 48 h, and 72 h. Differences between treatment in each cell line were compared, and statistical significance is indicated as *p* < 0.05 (*), *p* < 0.01 (**), and *p* < 0.001 (***). Lowercase letters represent differences in AhR induction as compared with the EC_50_ of TCDD in the respective cell line, a: significantly greater than TCDD *p* < 0.001, and b: effects significantly lower than TCDD *p* < 0.001.

To elucidate the capacity of the pool of colon microbiota-derived metabolites to induce the transcription factor AhR, we used cell-based in vitro reporter gene assays. Thus, three cell lines genetically engineered to detect AhR expression represented the potential interaction of metabolization products with the human host hepatic (HepG2), mammary (T47D), and intestinal (HT29) tissues. As shown in Figure 5, upon exposure to treatments, the AhR transactivity was significantly detected in T47D and HT29 for all except the inoculation day samples. Meanwhile, HepG2 cells treated with fermentation supernatant did not reveal any sign of AhR transactivation. The microbiota production of 15 common intestinal metabolites (Section 3.3) was statistically correlated with the AhR induction in HT29 cells (Exposure Dose 1) using Principal Components Regression (details in Supplementary File S1, Figure S1). 

## 4. Discussion

### 4.1. Microbiota Interindividual Diversity among Donors Was Pooled in the Fecal Inoculum

In the short-term simulation of the luminal microbiota conducted in this work, the fecal material was donated by three female healthy adults. The amplification of fecal DNA targeting the 16S rRNA gene allowed us to estimate the abundance of bacterial populations in the fecal inoculum. 

The microbial composition in the stools (see Figure 1) was characterized by an overrepresentation of Firmicutes phylum, which categorizes the donors as enterotype 3 (the most common one) [48]. However, enterotype 3 is commonly driven by a high abundance of the *Ruminococcus* genus [49], which was not really the case. A significant presence of *Coprococcus* characterized fecal samples from donor 1, which did not resemble donors 2 and 3. Meanwhile, the highly heritable *Christensenellaceae* [50], mainly represented by the genus *Christensenellaceae_R-7_group,* was found in high abundance in all donors (>6%), particularly donor 1, who also exhibited the highest abundance of *Bacteroides* (~9%). The UCG-002 genus from *Oscillospiraceae* family was found abundantly in all stool samples, but especially in donor 2 (12.5%). Similarly, *Oscillospirales_ge* was abundant mainly in feces from donor 2 (~9%), but also in donor 3 (6.3%). The family *Oscillospiraceae* has been negatively correlated with cholesterol levels in Mexican children suffering from obesity [51]. Furthermore, the presence of the *Oscillospiraceae* family in the stool is associated with lower extra-intestinal pain in women suffering from irritable bowel syndrome (IBS) [52]. A recent detailed analysis (in >6000 subjects) of *Oscillospira* has revealed this genus as a possible aggravating biomarker of constipation, suggesting a genera-specific role within this family in the human gut microbiota yet to be analyzed [53]. 

The relatively new sub-branch *Alistipes* genus from the Bacteroidetes phylum was identified in high abundance (~9%) only in donor 2. Dysbiosis of this population in humans appears to play either a beneficial or detrimental role. This is particularly important in the context of colorectal cancer and intestinal inflammatory conditions [54]. The genus *Phascolarctobacterium* was also exclusively identified in donor 2. Previous studies have demonstrated a high colonization rate of the *Phascolarctobacterium faecium* species in human feces [55]. However, only a few works are available on this genus, despite its potential correlation with the mood and metabolic state of humans [56]. 

The *Faecalibacterium* genus is an important population in human feces, and, within it, *Faecalibacterium prausnitzii* is one of the most abundant species in the human gut. This commensal bacterium is a butyrate producer and has a pleiotropic but mostly beneficial role in the gastrointestinal tract [57]. Meanwhile, the *Akkermansia* genus from the Verrucomicrobia phylum is frequently represented by *Akkermansia muciniphila* in the human intestinal microbiota. These two commonly found genera (i.e., *Faecalibacterium* and *Akkermansia*) are considered host health promoters. Each of them accounted for more than 5% abundance in all samples in this study. In donor 3, the *Akkermansia* genus accounted for more than 30% abundance, which is much higher than usually reported [58]. 

Ultimately, the gut microbial footprint of all three donors was highly consistent with a “core microbiota” (i.e., genera shared by 95% of samples) suggested from a large-scale study of fecal samples from the general population in Belgium and the Netherlands that included thousands of participants [59]. Although important inter-individual differences were identified between donors, this was expected, since the gut microbial community is different even for people who are closely related [60] or who have similar diet patterns [61]. Demographic factors such as age, gender, and ethnicity could also influence bacterial composition in human stool [62]. Indeed, the individual gut bacterial composition is distinctive, and it is difficult to establish patterns in its intrinsic diversity [63]. Furthermore, there is not a unique optimal microbiota composition [64], but there is a limited number of well-balanced hosts–microbial symbiotic states [48]. 

It is foreseeable, then, that microbial community reproducibility and stability in in vitro experiments are difficult to accomplish [65], particularly using fecal samples of a single individual. Henceforth, we used a pool of microbiota as a fermentation matrix. Among the advocated advantages of pooling fecal samples are the diversity and representativeness of the human colon ecosystem [37]. 

### 4.2. Use of a Convenient and Short-Term In Vitro Simulation of the Human Colon

Humans are holobionts and not autonomous entities, as used to be thought [66]. Therefore, disturbances in the microbial communities that inhabit us may lead to shifts from healthy to pre-disease and disease states [67]. Host-specific assemblages of microbes in the gut microbiota add complexity to the mechanistic study of its multiple functions. Further, accessing human colon sites is highly invasive and requires medical supervision. The use of in vitro human gut fermentation models provides a technological platform to overcome ethical concerns of in vivo and clinical experimentations. These models offer unique insights into gut microbiota function and, coupled with in vitro human intestinal cell models, molecular mechanisms of the host–microbe interplay can be addressed [68,69]. 

Compared to multistage continuous fermentation, batch culture models have the limitation of a lack of resemblance in simulating the interdependent functions in the human gut [70]. However, short-term gastrointestinal ecosystem simulations are particularly useful to quickly profile the metabolic activity of the gut microbiota in a convenient and inexpensive setup [71,72]. A common disadvantage highlighted in batch systems is the weakness to control microbial growth, which is completely dependent on the inoculation density and substrate depletion rate. Moreover, such microbial evolution is often derived from important pH variations not measured in most batch simulations [68]. 

In previous reports, the prebiotic effects of *trans*-galactooligosaccharides and inulin have been evaluated on batch models under the strict control of anaerobic conditions, temperature (37 °C), and pH (6.8–7.0) to mimic conditions of distal regions from the human large intestine for 48 h [72]. In another study, a pH-controlled (6.6–7.0) fermentation over 24 h has allowed for assessing the potential impact of iron on gut microbial growth and the metabolism [73]. In this work, a slightly longer (72-h) experiment was designed. The pH was maintained as encountered in the human distal colon between 6.6 and 6.9, like in most static short-term simulations of the intestinal microbiota. 

### 4.3. Shifts Observed in the Bacterial Community Composition following Inoculation

Once the fermenters were inoculated, the evolution of target taxa was assessed by qPCR analyses of the fermentation samples. The qPCR method is a convenient and reproducible technique that allows for mapping of the distribution of phylogenetically distinct bacteria with high sensitivity [40]. Thus, a relative profile of fifteen microbial communities in the batch fermentation was drafted, as presented in Figure 2a–o. 

In general, the microbial richness seemed to be affected upon inoculation. Similar effects have been observed in previous studies and underlined the need for control experiments to study the induced effects of different treatments due to the very probable loss of taxa during the experiment setup and simulated fermentation [65]. However, this is not the case here, since only untreated microbiota were analyzed. 

A diminution was observed during the colon experiment for the Firmicutes phylum (the most abundant phylum in the inoculum), as shown in Figure 2b. However, a lactic acid-producing bacteria (LAB) belonging to this phylum (the *Enterococcus* genus) had an exponential increase during the 72 h fermentation (Figure 2l). This important colonization of *Enterococcus* was very consistent in the three replicated colonic systems. Some strains belonging to the *Enterococcus* genus are considered negative biomarkers of gut health due to their association with inflammatory bowel disease (IBD) [74] and with outbreaks of nosocomial infections [75]. However, several strains from this genus are health promoters [76]. For example, they are recommended in the treatment of serum cholesterol (*E. faecium* M-74) [77], antibiotic-associated diarrhea (*E. faecium* SF68) [78], and irritable bowel syndrome (*E. faecalis* DSM 16440) [79]. Moreover, a very recent study of the newly isolated *Enterococcus lactis* strain JDM1 assessed its probiotic potential and good gastrointestinal tolerance [80]. 

Other LAB genera quantified in the colon microbiota (*Lactobacillus* and *Streptococcus*), behaved very differently from *Enterococcus*. On the one hand, *Lactobacillus* (Figure 2i) seemed to increase during only the first day of the colon simulation. On the other hand, *Streptococcus* presented a high increase in the microbiota for two days, and then significantly decreased by the third day (Figure 2m). The presence of the *Lactobacillus* genus in human colonic microbiota has been either positively or negatively related to health [81]. Some strains such as *L. reuteri* can greatly contribute to metabolizing tryptophan into indole derivatives, and thereby activate the AhR in the host’s epithelial barrier, as well as limit the colonization of opportunistic pathogens [82]. Meanwhile, members of the *Streptococcus* genus are anciently known to cause life-threatening infections. However, recent studies have suggested the potential probiotic uses of some species such as *S. salivarius* [83] and *S. thermophilus* [84].

Together, the previous observations relative to LAB growth tendency could indicate a significant change in the incubated ecosystem by the third day of static fermentation. This might be caused by the production of toxic metabolites and could justify limiting the batch culture to 48 h, as others have suggested [71,72,85]. 

Nevertheless, the significant increase observed in the *Bifidobacterium* genus (Figure 2j) and in *Bacteroides-Prevotella* (Figure 2n) advocates in favor of a longer (72-h) fermentation. Such increases were not significant, but very consistent at the phylum level. On the one hand, the Actinobacteria phylum had a relatively constant profile during the incubation, which increased on the third day, as the *Bifidobacterium* genus. Various members of Bifidobacteria are among the earliest colonizers of the human gastrointestinal tract [86], and the genus is the subject of increasing interest as a health promoter [87,88]. Moreover, the *Bifidobacterium* species is widely studied as a live component of the so-called functional foods, and it is included in diverse probiotic formulations commercially nowadays [89]. On the other hand, the Bacteroidetes phylum (Figure 2a), as *Bacteroides-Prevotella*, was augmented (compared to the fecal inoculum) only on the third day of fermentation. The *Bacteroides-Prevotella* genera are linked to some specific dietary habits and have core functions in the human microbiota [90,91]. Thus, the present work indicates that during the last 24 h of incubation, these important biomarkers found better conditions to grow.

Other bacterial communities belonging to the Firmicutes phyla such as Cluster XIVa and Cluster IV decreased during the in vitro batch experiment (Figure 2e,f, respectively), even though several genera included in these groups were found in high abundance in the fecal inoculum (Figure 1). Previous investigations into the SHIME system have revealed that these clusters are enriched in the mucosal environment and their abundance is lower in luminal content [92]. Bacteria included in Cluster XIVa and Cluster IV are common indicators of microbiota health, mainly associated with the butyrate-producing capacity [93]. Consistently, the butyrate-producing genus *Roseburia* (Figure 2h) decreased during the incubation. Butyrate-producing bacteria profiled through ButCoA (Figure 2g) did not reveal any important changes in the qPCR analysis. Including a mucin compartment in the experimental setup of this work could probably favor the simulated ecosystem for the growth of these bacterial groups.

The *Christensenellaceae* family (Figure 2k), found abundantly in the fecal material of the three donors, was not preserved during the colon fermentation. In addition, the highly abundant *Akkermansia* in the feces (Figure 1a), targeted through the most common species found in the human colon (*Akkermansia muciniphila*), significantly decreased during the 72-h experiment (Figure 2o). 

Finally, the Gammaproteobacteria class increased significantly in the batch system (Figure 2d). This is presumed to be due to the diversity that comprises it, which includes several potential pathogenic genera (e.g., *Salmonella*, *Pseudomonas*, *Klebsiella*) [94,95]. 

In summary, some microbiota shifts were identified during the fermentation, as expected. However, most of the target populations were stably maintained without a significant increase or decrease. This might indicate that relative stability of the fermentation environment is reached after 48 h in the static and short-term simulation of the human colon. 

### 4.4. Chemical Metabolites Profiling the Microbial Fermentation 

Various bioactive chemical molecules from food digestion, xenobiotics processing, or directly generated by the gut inhabitants drive the cell-to-cell communication in the gastrointestinal tract. In fact, some crucial metabolites are mainly (or exclusively) produced by microorganisms and released to the intestinal environment to act locally or systemically [96,97]. Signal transduction, energy production and storage, host immunity, and epigenetic regulation are among the crucial roles of gut microbiota metabolites [98]. In this study, the colon microbiota metabolic capacity was profiled by analyzing important molecules derived from processing dietary fiber (i.e., SCFA, BCFA) and amino acids (i.e., biogenic amines). 

SCFAs are the main metabolites produced in the colon over dietary fibers or nondigestible carbohydrate fermentation [99]. Fatty acids are chemotaxonomic markers of metabolic health, and their increased production in the colon is linked to the improvement of gut barrier function and a reduction in intestinal inflammation [100,101]. A low percentage of SCFA produced in the colon is excreted in the feces (~5%). In fact, when detected in stool, they could be a sign of gut dysbiosis and permeability, as well as a risk factor associated with various diseases [102]. Once produced, most SCFAs are absorbed by the colonocytes or else incorporated into systemic regulation through the portal vein into the liver. Systemic concentrations of SCFA are much lower than colonic concentrations. Thus, rather dissimilar systemic availabilities of acetate (~36%), propionate (~9%), and butyrate (~2%) have been observed by stable isotope technologies using ^13^C-labelled fibers [103].

The production of SCFA is shaped by the individual microbiota composition, which is largely determined by dietary patterns. In the colon of adults between 18 and 50 years old, the total concentrations of SCFAs are estimated to be from 64–105 mM [104]. Meanwhile, the BCFA concentrations commonly reach a maximum of 5 mM [104]. The SCFA and BCFA measurements in the present study concurred with these suggested values, as observed in Figure 3g,h, respectively. Moreover, the relative percentage of the most abundant SCFAs (i.e., C2, C3, C4) reached levels in line with previous reports of in vitro colonic simulations [72,105]. 

At a local level, SCFAs are the major energy substrates of colonocytes, and multiple beneficial roles are attributed to them in the intestine. For instance, butyrate is a well-known regulator of mucosal inflammation, transepithelial fluid transport, gut barrier functions, and motility [106]. Valerate, although less explored, seems to have an important role in the intestinal barrier and a synergistic effect with the rest of SCFA to regulate intestine homeostasis [107]. The presence of acetate and propionate, as major microbial fermentation metabolites, modulate the entire gut ecosystem. Propionate participates in the control of satiety signals and in lowering cholesterol, and propionate-producing bacteria might alter gut microbe-dependent T cell differentiation [108]. Acetic acid-producing bacteria have been positively correlated with colonic motility and absorption, and thus with constipation, as they control the transit rate of the small intestine and the water content of the stool [109]. 

Furthermore, the effects of SCFA reach far beyond the intestinal context. Thus, they have a role in cardio-metabolic health [110], the pathophysiology of obesity [111], and type 2 diabetes mellitus [112]. Cell-based molecular methods have shown that AhR signaling pathways are activated by SCFAs, being the inhibition of histone deacetylase (HDAC), a plausible associated mechanism [34].

On the other hand, amino acid decarboxylation by microbial enzymes leads to the production of biogenic amines, nine of which were quantified in this work. 

Polyamines (i.e., putrescine, cadaverine, spermidine, spermine) are ubiquitous and essential for growth and differentiation in the large intestine, and they participate in the immunomodulation of the human gut microbiota [113]. Their upregulation in the colon has been studied in animal models to suppress inflammation and improve longevity [114]. These small polycationic molecules are emerging players in bacteria–host interactions due to their core physiological functions, as well as their role in bacterial pathogenesis [115]. In the present work, putrescine was mainly detected during the first 24 h of colonic fermentation. The concentration values (>200 µM) were consistent with previous fecal culture fermentations [116]. The lack of significant quantities of spermidine and spermine suggested that putrescine was not used to produce these metabolites. Meanwhile, the biogenic amine cadaverine was largely produced during the 3-day fermentation (>300 µM) (Figure 4a). Some authors observed that the cytotoxicity threshold of cadaverine in intestinal cells is twice that of putrescine [117]. However, there is scarce information reporting the expected values of these amines in the human intestinal context.

Methylamines are directly related to gut microbiota metabolism [118]. The high levels of methylamine detected here at the end of the batch experiment (Table 1) could be a consequence of the microbial transformation of essential methyl donor nutrients. A low abundance of methyl-donor nutrients such as choline could also explain the high levels of *Gammaproteobacteria* detected in the system [61]. 

The quantified productions of the aromatic 2-phenylethylamine and tyramine are probably associated with the abundant presence of *Enterococcus* in the fecal fermented systems [119]. These biogenic amines are both considered ligands of the so-called trace amine-associated receptors (TAARs), whose outcome effects are deeply implicated with neurologic, cognitive, and psychiatric manifestations, including sensory perception and taste [120]. The TAARs are considered an emerging pharmacological target for the treatment of human disorders [121]. Tyramine and 2-phenylethylamine are presumed to exhibit direct effects on human gut epithelial cells through TAARs modulation [122]. Meanwhile, bacteria-derived production of the heteroaromatic neurotransmitter histamine was possibly below the quantification limit (<8.4 mM) of the analytical method used.

Tryptamine (Figure 4b), derived from tryptophan metabolism, was detected throughout the whole fermentation process. Tryptamine, as well as its downstream metabolite indole acetic acid, are considered among the main tryptophan-derived ligands of AhR [123]. Interestingly, tryptamine is a CYP1A1 substrate, in line with the suggestion that AhR endogenous ligands are also regulated by CYP enzymes [124]. 

In general, the metabolism profiled through the production of distinct molecules in the static in vitro model allowed us to extract some relevant insights into the fermentative process in this class of experiment. First, the SCFA and BCFA, the aromatic amines tyramine, 2-phenylamine, and tryptamine, as well as the aliphatic cadaverine, are stably produced and detectable during the 72 h of the experiment. Therefore, this is probably a convenient and adequate setup to study these metabolites. Conversely, the production of some other aliphatic amines such as methylamine, spermine, spermidine, and putrescine might require no static but long-term fermentation models. 

### 4.5. AhR Transcriptional Activation Representing Host-Microbe Interaction 

Host–microbe interaction in the human gastrointestinal tract is a complex dynamic involving multiple pathways. Among these is the activation of xenobiotic sensors such as AhR. This receptor processes signals from endogenous and exogenous chemicals that trigger important molecular pathways through the induction of its transcriptional activity [125]. AhR signaling has been associated with the regulation at different levels of the immune response of the host organism, and it is considered a key element in the maintenance of homeostasis in the gut microbial ecosystem of mammalians [126,127]. 

The human colorectal adenocarcinoma HT29 is a cell model successfully used in the study of host–microbiome interactions, as it can express characteristics of mature intestinal cells [128]. There are several advantages of HT29 compared to the Caco-2 cell line (also derived from human colorectal adenocarcinoma) when studying the effects on the gut mucus layer [128,129]. HT29 culture models have been used, for example, to evaluate the impact of colonic fermentation of prebiotic flours (e.g., rye, lentils, wheat) [130], and the protective effects of probiotics (e.g., *Streptococcus thermophilus*, *Lactobacillus acidophilus*) [131]. In this study, the HT29-Lucia cell line was utilized to identify the potential activation of AhR transactivity caused by microbial-derived metabolites in the colonic environment. However, several gut microbial-derived metabolites could reach the liver or be incorporated into the systemic circulation, modulating health and disease through AhR [10,132]. Therefore, the potential activation of AhR transcriptional activity was also evaluated outside the intestinal context through HepG2 and T47D human cell models (Figure 5). 

The results obtained revealed that the metabolic output of the healthy human microbiota fermentation is able to induce AhR transactivity in intestinal cells (HT29) two-fold times higher than the well-known AhR agonist TCDD (Figure 5). Meanwhile, T47D cells were even more sensitive to the fermentation supernatant, with an AhR activation above 20-fold response by the second and third day of fermentation.

The induced activation of AhR transcriptional machinery in the intestine is probably caused by a synergistic effect of several metabolites. For example, indole derivatives (e.g., Indolo[3,2-b]carbazole (ICZ), 3,3′-diindolylmethane), arachidonic acid metabolites (e.g., Lipoxin 4A, Prostaglandin PGG2), heme-derived metabolites (e.g., bilirubin, biliverdin), and tryptophan metabolites (e.g., kynurenine, tryptamine, 6-formylindolo[3,2-b]carbazole (FICZ)) are known endogenous ligands of the AhR derived from diet, photo-oxidation, host or microbiota metabolisms, indistinctly [30].

Mechanistic studies have shown how the AhR transcriptional activity induced by tryptophan metabolites is crucial to maintaining the fragile equilibrium between the microbiota and the host cells [133]. Thus, tryptophan catabolites derived from gut microbiota are dysbiosis biomarkers, and their production is a commonly suggested pharmacological target [14,15,82,134].

Tryptamine, as previously mentioned, was stably produced during the 72-h experiment, and it is a long-known AhR agonist [135]. The first studies of tryptamine-induced activation of AhR suggested that effective tryptamine concentrations were between 80–100 µM [123]. However, it has been later revealed in cell models that tryptamine can induce CYP1A1 gene expression even at very low concentrations (~5 µM) [136]. Therefore, the concentrations of tryptamine detected in the human microbiota herein modeled (10–30 µM) probably contribute to the AhR activation displayed by the metabolic output (Figure 5).

Additionally, SCFAs are probably major contributors to the AhR-induced activity, since they have all proven to be AhR modulators. Thus, at similar concentration levels to those found herein, other authors have shown that butyrate (~10 mM), acetate (~40 mM), and propionate (~20 mM) can induce AhR-responsive genes in vitro. The most significant AhR activity has been shown for butyrate, which has led to the suggestion of this metabolite for reducing systemic autoimmune disorders such as rheumatoid arthritis [34].

Interestingly, the AhR activity displayed by the metabolic output was not greatly increased with the higher exposure dosage (Figure 5b). Possibly, the metabolic mixture produced by the intestinal microbiota is able to maintain a balanced and self-regulated induction of AhR transcriptional activity [137]. Indeed, an exacerbated cellular response of AhR such as the one exerted by persistent organic pollutants, and particularly dioxin-like compounds, is associated with the triggering of toxicity pathways and affects the transcriptome in a distinct way [138]. Meanwhile, transient but commonly potent AhR activation is induced by endogenous ligands such as the well-studied tryptophan derivative FICZ [139]. This last one is probably the form of interaction of microbial metabolites with AhR due to the physiological machinery regulating their production and degradation in the biological context. In fact, potential drugs through microbial metabolite mimicry have been recently suggested as AhR agonists with proven in vivo therapeutical use in intestinal inflammation [140].

Finally, the results of principal component regression revealed a possible correlation between the metabolic profile of the fermentation process and AhR transactivation at the lowest exposure dose (Figure S1). However, further experimental corroborations are needed.

## 5. Concluding Remarks and the Future Ahead

Analyzing the evolution of healthy human colon microbiota across several targeted bacterial populations allowed us to delve into some interesting positive and negative biomarkers of health and disease. This is particularly important as it is suggested that the “healthy microbiome state” is a spectrum of numerous states in a delicate balance, which can only be addressed by studying various markers [141].

There were certain limitations of the human colon fermentation model to maintaining and promoting a favorable growth environment during 72 h for certain taxa. Evaluating the composition of the microbes adhered to the intestinal mucosa could be a future added endpoint. Thus, incorporating mucosal environments could contribute to more representative colonization of some communities such as *Clostridium* cluster XIVa [92] and *Lactobacillus* [142]. However, in a recent short-term colonic fermentation used to study probiotic ingredients, no major differences were observed between incubations with or without a mucosal environment [143].

On the other hand, although several authors have suggested no significant overall difference in female and male gut microbiota, especially in the case of healthy individuals [144,145,146], gender-related differences could be an important factor to be considered [147]. Nevertheless, in previous studies, a greater α-diversity was found in the gut microbiota composition of females [148,149,150].

Future research directions of this work should also lead to a comprehensive characterization of the metabolic production during colonic fermentation. Individual metabolites and their mixtures should be tested at realistic concentration levels to assign their contributions to the activation of AhR transcriptional activity.

## Figures and Tables

**Table 1:** Biogenic amines produced during the in vitro colonic simulation.

Class	Amines	Structure	0 h	24 h	48 h	72 h
Aliphatic amines	Methylamine	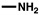	-	-	35.75 µM ^†^	283.86 µM ± 128.76
	Spermidine	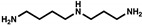	0.75 µM ^†^	-	0.12 µM ^†^	-
	Spermine	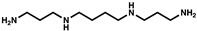	-	-	-	-
Aromatic amines	2-phenylethylamine	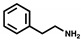	-	5.20 µM ± 0.96	3.13 µM ± 4.38	4.58 µM ± 1.62
	Tyramine	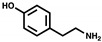	-	27.42 µM ± 19.87	25.42 µM ± 20.79	28.85 µM ± 7.93
	Histamine	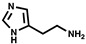	7.51 µM ^†^	-	-	-

^†^ Only detected in one replicate, (-) below the limit of quantification (LOQ).

## Data Availability

Data is contained within the article or supplementary material.

## Supplementary Materials

The following supporting information can be downloaded at: https://www.mdpi.com/article/10.3390/foods11131946/s1, Figure S1: Principal components regression between the metabolic profile of the fermentation process and AhR transactivation at the lowest exposure dose (Dose 1), Table S1: Sequence and annealing temperature used for each taxon-specific qPCR experiment. Table S2: Metagenetic analysis. References [151,152,153,154,155,156,157] are cited in the supplementary materials.
