# Supplementary material for: Human Adult Microbiota in a Static Colon Model: AhR Transcriptional Activity at the Crossroads of Host–Microbe Interaction
Source: Foods. 2022 Jun 30;11(13):1946. doi: 10.3390/foods11131946 (PMC9265634; doi:10.3390/foods11131946)
Supplement: Supplementary file 1 [file foods-11-01946-s001.zip › Supplementary File S1.pdf]

Article

Human Adult Microbiota in a Static Colon Model: AhR Transcriptional Activity at the Crossroads of Host–Microbe Interaction

Elizabeth Goya-Jorge [1], Irma Gonza [1], Pauline Bondue [2], Caroline Douny [3], Bernard Taminiau [4], Georges Daube [4], Marie-Louise Scippo [3], and Véronique Delcenserie [1,*]

[1] Laboratory of Food Quality Management, Department of Food Sciences, Faculty of Veterinary Medicine, University of Liege, Av. de Cureghem 10 (B43b), 4000 Liege, Belgium; egoya@uliege.be (E.G.-J.); iegonza@uliege.be (I.G.)

[2] Research & Development, ORTIS S.A., Hinter der Heck 46, 4750 Elsenborn, Belgium; pauline.bondue@ortis.com (P.B.)

[3] Laboratory of Food Analysis, Department of Food Sciences, Faculty of Veterinary Medicine,
University of Liege, Av. de Cureghem 10 (B43b), 4000 Liege, Belgium; cdouny@uliege.be (C.D.); mlscippo@uliege.be (M.-L.S.)

[4] Laboratory of Microbiology, Department of Food Sciences, Faculty of Veterinary Medicine,
University of Liege, Av. de Cureghem 180 (B42), 4000 Liege, Belgium; bernard.taminiau@uliege.be (B.T.); georges.daube@uliege.be (G.D.)

* Correspondence: veronique.delcenserie@uliege.be; Tel.: +32-4-366-51-24

Section 2. Material and Methods

Table S1. Sequence and annealing temperature used for each taxon-specific qPCR experiment

Nº	Target taxon (ID)	Primer sequence	Tann [a] (This study)	References [b]
1	Universal bacteria	F: AAA-CTC-AAA-KGA-ATT-GAC-GG<br>R: CTC-ACR-RCA-CGA-GCT-GAC	61.5 ºC	[41]
2	Bacteroidetes phylum	F: CRA-ACA-GGA-TTA-GAT-ACC-CT<br>R: GGT-AAG-GTT-CCT-CGC-GTA-T		
3	Firmicutes phylum	F: TGA-AAC-TYA-AAG-GAA-TTG-ACG<br>R: ACC-ATG-CAC-CAC-CTG-TC		
4	Actinobacteria phylum	F: TAC-GGC-CGC-AAG-GCT-A<br>R: TCR-TCC-CCA-CCT-TCC-TCC-G		
5	Gammaproteobacteria class	F: TCG-TCA-GCT-CGT-GTY-GTG-A<br>R: CGT-AAG-GGC-CAT-GAT-G		
6	Clostridium coccoides (Cluster XIVa)	F: AAA-TGA-CGG-TAC-CTG-ACT-AA<br>R: CTT-TGA-GTT-TCA-TTC-TTG-CGA-A	61.5 ºC	[151]
7	Clostridium leptum (Cluster IV)	F: GCA-CAA-GCA-GTG-GAG-T<br>R: CTT-CCT-CCG-TTT-TGT-CAA		
8	ButCoA	F: TGG-ACA-GAA-AGG-TTG-CGG-AG<br>R: GTG-TGT-ACG-CCC-AGA-TCC-TT	60 ºC	-
9	*Bacteroides* and *Prevotella* genera	F: GAG-AGG-AAG-GTC-CCC-CAC<br>R: CGC-TAC-TTG-GCT-GGT-TCA-G	54 ºC	[152]
10	*Roseburia* genus	F: GCG-GTR-CGG-CAA-GTC-TGA<br>R: CCT-CCG-ACA-CTC-TAG-TMC-GAC	61.5 ºC	[153]
11	*Lactobacillus* genus	F: CAT-CCA-GTG-CAA-ACC-TAA-GAG<br>R: GAT-CCG-CTT-GCC-TTC-GCA	62 ºC	-
12	*Bifidobacterium* genus	F: GGG-TGG-TAA-TGC-CGG-ATG<br>R: CCA-CCG-TTA-CAC-CGG-GAA	54 ºC	[154]
13	*Christensenellaceae* family	F: GYT-CGC-GTC-CCA-TTA-GVT-AGT-TGG<br>R: CAC-GTA-GTT-AGC-CGG-RGC-TTC-CTC	61.5 ºC	[155]
14	*Streptococcus* genus	F: AGA-GTT-TGA-TCC-TGG-CTC-AG<br>R: GTT-AGC-CGT-CCC-TTT-CTG-G	54 ºC	[156]
15	*Enterococcus* genus	F: CCC-TTA-TTG-TTA-GTT-GCC-ATC-ATT<br>R: ACT-CGT-TGT-ACT-TCC-CAT-TGT	61.5 ºC	
16	*Akkermansia muciniphila* species	F: CAG-CAC-GTG-AAG-GTG-GGG-AC<br>R: CCT-TGC-GGT-TGG-CTT-CAG-AT	54 ºC	[157]

[a] Annealing temperatures (Tann), [b] Protocol conditions from the references in all cases were re-validated in this study.

Section 3. Results: Principal Components Regression

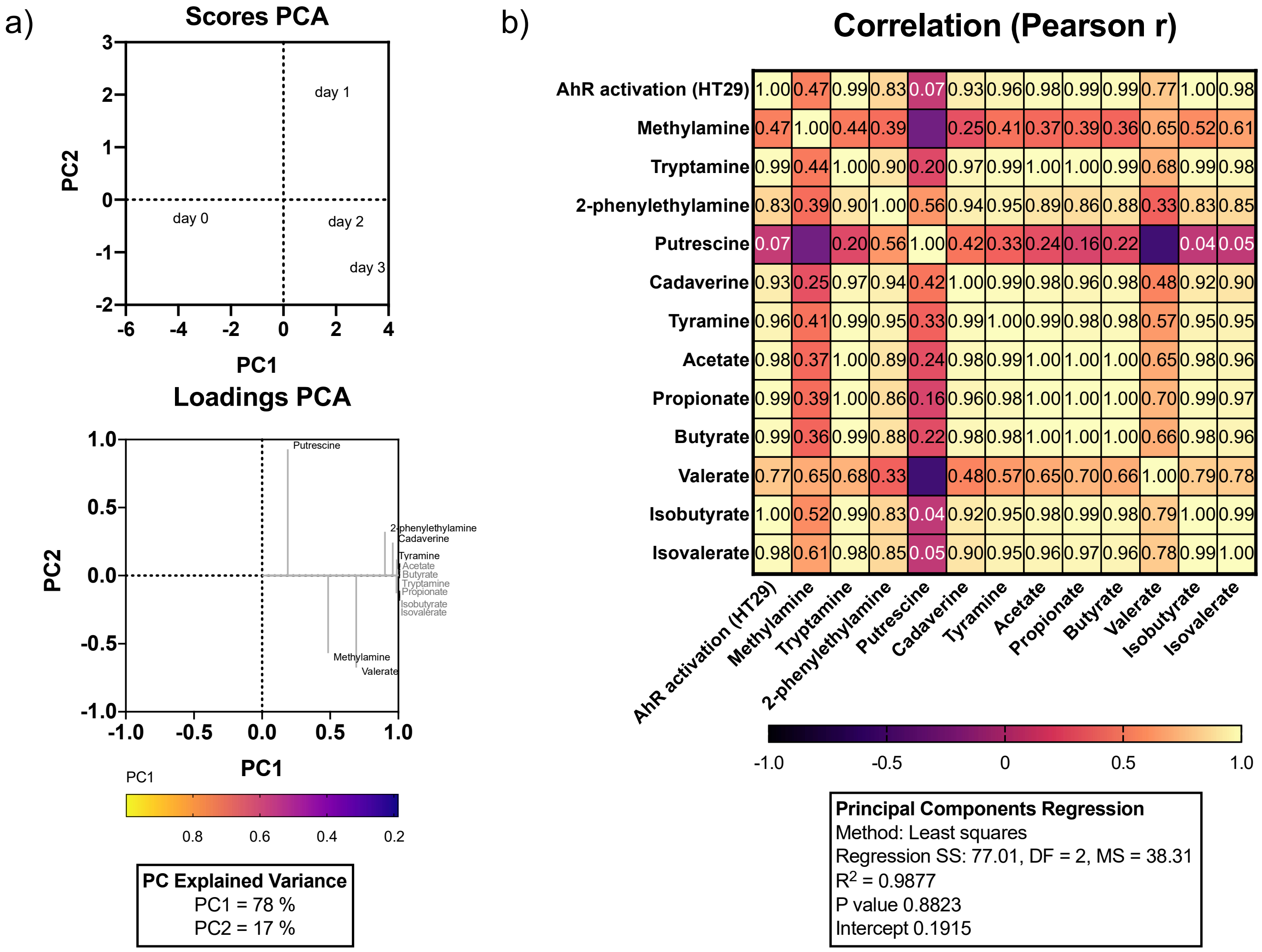

Figure. S1. Principal components regression between the metabolic profile of the fermentation process and AhR transactivation at the lowest exposure dose (Dose 1).
